# Introducing waterbirth in a university hospital setting in Sweden: A qualitative study of midwives’ experiences

**DOI:** 10.18332/ejm/188193

**Published:** 2024-06-03

**Authors:** Karin Larsson, Malin Bogren, Hanna Ulfsdottir

**Affiliations:** 1 Labor and birth department, Sahlgrenska University Hospital, Gothenburg, Sweden; 2 Institute of Health and Care Sciences, Sahlgrenska Academy, University of Gothenburg, Gothenburg, Sweden; 3 Department of Women's and Children's Health, Karolinska Institutet, Stockholm, Sweden

**Keywords:** waterbirth, midwives, focus group interviews, promoting factors, obstructing factors, high-income country

## Abstract

**INTRODUCTION:**

Waterbirth is a popular and increasing care option in several countries but is still debated. In Sweden, there are challenges in the process of reintroducing waterbirth after decades of interruption invoked by a dissuasion. The aim of this study was to explore factors affecting midwives’ provision of waterbirth at a university birthing clinic in Sweden.

**METHODS:**

A qualitative research design was used with three focus group interviews with 18 midwives at three birthing units. The data were analyzed using the principles of inductive content analysis.

**RESULTS:**

The midwives in the study expressed positive attitudes and potentiality about waterbirth, contributing to their desire to support physiological birth. However, obstacles were also disclosed, maiming waterbirth evolvement. Hence, two categories emerged, promoting factors and obstructing factors. The subcategories were: Provides a good experience whilst promoting physiological birth; Increased knowledge and information about waterbirth; Support from management; Updated guidelines; Ergonomic challenges; Lacking practical conditions; Lack of knowledge; Paradigm conflicts; and Limiting guidelines.

**CONCLUSIONS:**

The study concluded that midwives recognized both promoting and obstructing factors affecting the provision of waterbirth. The predominant factor highlighted was the care-culture, with a clear distinction between a risk-focused, medicalized approach that inhibits waterbirth and a salutogenic perspective advocating for it. This dichotomy underscores the importance of providing opportunities that support women’s choices to facilitate an empowering birth experience.

## INTRODUCTION

Waterbirth is a birthing method that has gained popularity in recent years due to its perceived benefits and endorsement of physiological birth^1-4^. Research has shown waterbirth to be a safe option for women and newborns when studying clinical outcomes, alongside its favorable impact on the birthing experience for women^5-7^.

Moreover, studies suggest that waterbirths involve fewer interventions compared to conventional births, facilitating the desired goal of physiological birth particularly valued by midwives^1,8,9^. Pregnant women are increasingly seeking options that prioritize physiological birth, making waterbirth a method that responds to this demand^10^.

Studies have shown that the relaxing environment provided by bath suites in birthing clinics and the pain-reducing effects of water immersion can lead to increased oxytocin production, which in turn can result in a beneficial contraction pattern and a birth with fewer interventions and complications^11,12^. Endogenous oxytocin can pass the blood–brain barrier and promote bonding with the newborn^13^. Therefore, the availability of water immersion during childbirth should be offered to a high extent at maternity units to improve the possibilities for women to have a physiological birth^11^. Additionally, beyond the physical benefits and safety of waterbirth, it has been shown to improve the birth experience and contribute to an empowering experience for women^3,7,14-16^.

However, its use has been a topic of debate in Sweden since the 1990s, when the National Board of Health and Welfare issued a dissuasion against waterbirth due to lack of evidence. This resulted in the discontinuation of waterbirth practice in Swedish maternity clinics, and midwives’ knowledge of the technique was hindered for decades. With the increasing request for waterbirth from pregnant women, Swedish maternity clinics are now facing challenges in re-implementing the practice while ensuring adherence to clinical guidelines and maintaining patient safety. In 2022, 23 out of 46 birthing clinics in Sweden offered waterbirth, and more clinics are at a starting point to introduce the birthing method.

Nonetheless, because of diminished experience and a persistent skepticism from some midwives and obstetricians, the re-implementation process has met with obstacles^17^.

In 2019, the first set of guidelines regulating waterbirth was introduced within a large maternity clinic located in the western region of Sweden^18^. However, these guidelines were highly detailed, and risk oriented which created barriers for many women who desired waterbirths. It is well known that midwives play a crucial role in facilitating physiological births, yet their actions and practices are influenced by the ward culture and local guidelines^4,19^. Restrictive guidelines may hinder midwives’ ability to assist waterbirths and limit women’s autonomy and decision-making^20,21^. Considering this, the aim of this study was to explore factors affecting midwives’ provision of waterbirth at a university birthing clinic in Sweden.

## METHODS

### Design

This study used a qualitative research design with focus group interviews with midwives working at three birthing units in western Sweden. Qualitative research design was considered the most useful to answer the research question: ‘What are the factors influencing midwives in offering waterbirth?’.

### Setting

The maternity care in Sweden is publicly funded, and most of the births are conducted at hospitals where the care is characterized by high levels of medicalization. Less than 1% of the births are planned homebirths, and only a few of those are publicly funded. Midwives are primarily responsible for normal births, while obstetricians are consulted in case of complications.

The University Hospital in western Sweden is one of the largest units in Northern Europe, facilitating 10000 births each year. There is no continuity of care as women are randomly assigned to a midwife upon arrival. Prenatal maternity care is provided by midwives in the community. During births, women are admitted to the hospital, where a separate team of midwives provides care. Postnatal care takes place by a team comprising nurses, auxiliary nurses, and midwives. The hospital comprises three units, one of which caters to high-risk births, while the other two provide standard care. All three units offer waterbirth, and the hospital has a total of five bathtubs.

### Participants

The study consisted of a convenient sampling of 18 midwives employed at the three birthing units. Participants were recruited through staff information letters sent by email. Participants signed up for one of the three interview sessions that best suited them. All participants had experience of attending waterbirths. A few held managerial, developmental roles, or research roles, all involved in the re-introduction of waterbirth at the clinic in different ways. The age of the participants ranged from 30 to 62 years (mean: 44 years), most of the participants worked with labor and birth. Fictitious names were assigned to the participants’ quotes.

### Data collection

The midwives were divided into three focus groups, with 5–7 participants respectively. The interviews were all conducted by the first author (KL) and took place at the hospital in a meeting room the first quarter of 2022. From being employed at the clinic, KL knew some of the participants, but no special effort was made to establish a relationship prior to the interviews.

An interview guide was developed with semi-structured questions and used as a support to the researcher in keeping structured conversations with equivalent content in all interviews^22^. It was constituted of a commencing question: ‘What comes to your mind when you hear the word waterbirth?’. Remaining questions were formed to direct the discussion to answering questions about factors affecting waterbirth practice. The interview guide was successfully pilot tested at the first interview and no alterations were needed; hence, this interview was included in the analyses. The interviews had an average duration of 44 (range: 39–47) minutes.

### Ethics statement

Participating midwives signed a consent form before taking part in the focus group interviews, receiving information about the study and the option to withdraw from participation at any time. The management of the hospital where the study was conducted approved the study and its publication. This study was approved by the Swedish Ethics Review Authority (reference number 2022-00040-01).

### Data analysis

The interviews were recorded and transcribed verbatim. The transcripts were analyzed using the principles of inductive content analysis as described by Graneheim and Lundman^23^. First, the text was read several times to make sense of the text as a whole. Next, meaning units were identified which answered the research question: ‘What factors affect midwives in providing waterbirth?’. The meaning units defined as words, sentences or paragraphs, were identified, and condensed. The meaning units were then compared and sorted into codes based on similar content, which were thereafter compared and clustered into sub-categories, which were sorted into categories. Throughout the analyses, the original transcripts were referred to several times to ensure that the results reflected the whole, and maintained the validity, of the text. The analysis was conducted by KL, in close collaboration with HU, until full agreement was reached. The final phase of the analysis involved all three authors. An example of the analytical process is shown in Table 1.

**Table 1:** Example of the analytical process

*Meaning unit*	*Code*	*Subcategory*	*Category*
*‘It’s a comforting and safe ambience and a recollection to support normal birth physiology. Many times it’s a welleducated woman who believes in her own capacity to give birth. She believes in her body and is less needy of our support than a woman giving birth in a normal room. She does it herself, and we can just step aside, and let her do it. It’s very powerful!’* (FGD 3)	Empowering mode of birth	Provides a good experience whilst promoting physiological birth	Promoting factor
*‘But as a student you hear absolutely nothing about it. And then it really depends on the supervisor you meet in clinical training, if she doesn’t show you then there is a much lower chance that you will start assisting waterbirths as a newly qualified midwife.’* (FGD 1)	Insufficient education in midwifery syllabus and clinical training	Lack of knowledge	Obstructing factor

## RESULTS

Factors affecting the use and implementation of waterbirth were sorted into two generic categories with respective subcategories. An overview is shown in Table 2.

**Table 2:** Categories and sub-categories describing the use and the implementation of waterbirth

*Categories*	*Sub-categories*
**Promoting factors**	Provides a good experience whilst promoting physiological birth
	Knowledge and information
	Support from management
	Updated guidelines
**Obstructing factors**	Ergonomic challenges
	Lacking practical conditions
	Lack of skills and knowledge
	Paradigm conflicts
	Limiting guidelines

### Promoting factors

*Provides a good experience whilst promoting physiological birth*

Compared to conventional births, waterbirth was described as a positive peaceful experience for both the woman and the midwife, with fewer interventions and greater possibilities of supporting the natural birth process by means of watchful attendance, and by ‘being’ with the woman instead of ‘doing’ things to her.

The midwives’ descriptions of waterbirth included expressions such as calm, peaceful, beautiful and cozy. The midwives claimed that the water’s ability to provide buoyancy effect was an advantage and the pain relief offered by the warm water was a good natural comfortable method.

Sharing such a birth was described as an extraordinary experience that correlated well to the midwives’ view of birth, supporting the woman’s own capacity and promoting empowerment. Midwives who were comfortable with waterbirths felt the urge to spread the practice to more midwives, thus making it more available:

*‘It was amazing to assist my first waterbirths. I know that I felt … it was so fantastic. I could not believe birth could be like that. I felt I wanted to offer it to more women, yes it was a very special experience. As a midwife you are dependent on how the birth is for the woman. I mean that is our greatest goal, to help women achieve an empowering birth experience.’* (Maria, FGD 2)

The opinion that waterbirth leads to less interventions during labor and a more salutogenic approach, was apparent in the interviews. Many of the midwives believed that waterbirth was a necessary counterbalance to a medicalized mode of birth care. The midwives obtained a more relaxed feeling at birth, with a greater trust in the birthing process with a reduced risk focus:

*‘*A*s a midwife I oddly enough have greater faith in women’s bodies and in the birthing process when the woman is immersed in water. I also become more relaxed.’* (Linda, FGD 1)

The cozy environment in the bath suite contributed to the positive experience, and assets such as star lightning, wall decorations, plants and calm music ensured a relaxed atmosphere in the room which made the midwives feel more at ease with the birth process.

*Knowledge and information*

Knowledge was described as important not only for the midwives at the birthing units, but also for the women, their partners, and the prenatal midwives. The midwives believed it was of great importance that the women were informed about waterbirth prior to birth.

Since midwives are the only providers of waterbirth, they valued their skills highly and requested frequent workshops and updates. Discussions at rounds were brought up, to spread knowledge also to the obstetricians and the assistant nurses. They suggested short reflections after waterbirths to further improve communication in the team, and give and receive feedback.

Social media were considered an important information channel to pregnant women. The families could see what the bath suits looked like, acquire information about giving birth in water, about guidelines, and practicalities were explained in a lucid way on the Instagram account presenting the clinic. As a result, women’s awareness of waterbirth was enhanced and a growing number of women requested waterbirth in their birth plan, putting pressure on the midwives to incorporate waterbirth into their practice.

The midwives felt a responsibility to teach midwifery students about waterbirth. They were determined to pass on the legacy of midwifery and physiological birth, where waterbirth was an important part. To share the elated feeling of a waterbirth with a student meant sharing the core of midwifery:

*‘I’ve included a lot of students in the waterbirth practice. And I have great experiences of it. Really amazing experiences! We’ve had a lot of fun. It’s something very special.’* (Leona, FGD 3)

Theoretical teaching included in the midwifery curriculum and practical workshops in the student’s clinical training were requested. As more midwives start to assist waterbirths more students will also be eligible to the practice, creating a positive spiral of knowledge. Peer teaching was important in introducing colleagues in a safe and positive manner, the assisting midwife could see how the birth was managed in regard to fetal monitoring, perineal support, birth positions, communication with the woman, delivering the placenta, estimating postpartum bleeding, among other factors. They claimed it to be especially important to invite midwives that did not yet have experience of waterbirth in order to spread the practice:

*‘And I think you build like a bank of experience. It takes time, but the more you subject yourself to it the more the procedure is normalized. Once you have been second midwife at a few births you might feel like, alright, now I can do this myself. It’s a process that takes time.’* (Rosa, FGD 3)

During this research, workshops have begun at the clinic. The interviewed midwives were very positive, and they felt safer, more informed and had a new sense of enthusiasm which increased their willingness to assist waterbirths.

*Support from management*

Support from the management was an important factor in the implementation of water births. The managers’ ability to organize workshops and influence the birth culture enabled them to support the process. Their financial responsibility empowered them to decide about purchase of bathtubs and utilities which were thought to have a big impact on the care, supporting a salutogenic approach to birth, compared to a more medicalized focus. If the managers were positive and supportive it affected the attitudes of the staff. After introducing guidelines, midwives perceived that the managers’ attitudes towards waterbirth became more positive:

*‘That’s really what you need. Support from the ward. The problem is not to assist the woman in the tub. If you feel the support from the management, it makes you feel safe and not questioned.’* (Anna-Lena, FGD 1)

*Updated guidelines*

After guidelines were updated with less regulations, it was also a sign from the hospital that waterbirths were no longer considered as risky and controversial. The midwives felt relieved, with a greater sense of being able to work according to midwifery values, and to follow the woman and her wishes for a physiological birth:

*‘I have greater opportunities now to offer waterbirth with more allowing guidelines. Now guidelines have more focus on the woman’s wishes for her birth and her health factors and don’t just focus on every single risk factor.’* (Linda, FGD 1)

### Obstructing factors

*Ergonomic challenges*

There were different opinions about waterbirth in relation to ergonomics and some believed it caused back-problems. For some it was a significant hindrance, while others did not see it as a concern, instead they regarded it acceptable in order to follow the woman’s desire to birth as she desired. With the woman submerged in the tub, perineal protection was perceived as challenging as the hospital recommends hands-on protection for all vaginal births:

*‘Yes it’s difficult. I raised and lowered the bathtub. But I have to get a clear view of the perineum and have to be there and bend over the tub. It hurt my back. But I heard you don’t have to be there so much, that the water protects the perineum, but I’m not comfortable with that, just to stand there and not hold manual protection when the baby comes.’* (Solveig, FGD 2)

*‘That’s the essence of being a midwife. You have to adjust. That’s the charm and the art of our profession. You just have to find something that works for you.’* (Helena, FGD 2)

Intermittent monitoring and vaginal examinations were also practices that were described as more difficult in waterbirths in regard to ergonomics.

*Lacking practical conditions*

In the effort to scale up the waterbirth practice, several flaws were identified in regard to practical conditions. For example, there was a shortage of bathtubs, limiting the opportunity for women to immerse in water. There were also concerns about the hot and humid ambience in the room and the exposure to dirty bathwater. The protection equipment offered was not perceived to fully protect the midwives. They could see that the workload and lack of time were obstructing factors. The increased number of births with risk profiles were also named as obstacles:

*‘There are so many medicalized births now. Oxytocin infusions and epidurals, fevers and such. We induce so many women. Women who would like a waterbirth are excluded and don’t have the chance for that option. We cannot offer one-to-one care, and the support for the woman is not there and instead they choose an epidural.’* (Hanna, FGD 1)

*Lack of skills and knowledge*

There was a consensus in the group that knowledge about the procedure and of guidelines was deficient among prenatal midwives. The midwives expressed concern and requested waterbirth to be compulsory basic knowledge:

*‘It is quite person based then, since some midwives aren’t comfortable with the concept. Then it depends on which midwife the woman meets, you know if waterbirth is even possible. And it’s good, as the implementation process continues, that more midwives suggest it, that it’s something we offer here.’* (Karin, FGD 2)

Some of the midwives expressed a feeling of insecurity concerning the practical management of waterbirth, for instance in case of emergencies or complications. This made some midwives hesitant to offer waterbirth as they felt safer to handle complications on land.

*Paradigm conflicts*

The most evident obstacle that came forth in the interviews was the negative attitudes towards waterbirth coming from the obstetricians. The midwives felt obliged to defend their waterbirth practice as obstetricians expressed a skeptical view causing midwives to feel questioned, and friction was evident in many situations. The obstetrician’s hesitant approach made the support of the midwifery corps and the support of the managers crucial.

The increasing focus on teamwork raised uncertainty about the professional boundaries between midwives and obstetricians. There was a notion that obstetricians wanted to control the work of midwives and claim normal births as part of their responsibility:

*‘What we associate with normal birth and midwifery can cause fear in other professions, making it a barrier. And whether it is at all possible to achieve depends on the prevailing structure. Whose knowledge rules? And how much power do our respective professions skills have?’* (Sofia, FGD 3)

Midwives experienced a shift in focus in waterbirth when discussed at rounds, from a salutogenic low-risk approach to one of risks. Hierarchical structures emerged when midwives felt compelled to follow obstetricians’ directives. There was a belief that the obstetricians not only had the power, but also wanted, to restrict the use of waterbirth and in the long run, ban it altogether.

Some midwives involved obstetricians as little as possible to avoid disruptions in the waterbirths, while others believed it was advantageous to invite them in the process, taking part at the waterbirth workshops recently initiated by the hospital and to be present at the births:

*‘If you do something behind locked doors and don’t invite them there will be resistance. We want a strong support from the medical profession in developing normal birth. And in that respect I see no other way than inviting them in there, in case they want to join.’* (Sofia, FGD 3)

The midwives expressed a fear that involving obstetricians would affect midwives’ field of work and change the concept of waterbirth into a more medicalized and risk-focused matter, increasing the use of interventions. Midwives believed medical reluctance evolved from fear, lack of knowledge, and unfamiliarity, limiting their ability to have control of those births and of the midwives’ work, compared to conventional births.

*Limiting guidelines*

Before guidelines were in place, waterbirths were handled with discretion, rarely documented in the patients’ records. Midwives were mostly positive to the implementation of the guidelines; they eliminated the sense of breaking rules. Fear of adverse outcomes in the bath was mitigated when guidelines approved the practice. There was a fear of jeopardizing waterbirth if there was a bad outcome:

*‘You think … is this allowed? Is it legitimate? Am I working within my boundaries? That’s why guidelines are important. It states that its legitimate birth practice. And even if something was to happen during the birth … well if you followed guidelines at least you did nothing wrong.’* (Camilla, FGD 2)

There was frustration about denying women waterbirth because of limitations in the guidelines. Midwives had a strong desire to fulfil women’s wishes and helping women to achieve a positive birth experience. There were moments of conflict when this desire could not be fulfilled, because guidelines prohibited some women from having a waterbirth.

## DISCUSSION

This study from Sweden revealed both promoting and obstructing factors affecting the use of waterbirth. Promoting factors for offering waterbirth included enhancing the birthing experience and supporting physiological birth, backed by knowledge, managerial support, and updated guidelines. Conversely, obstructing factors encompassed ergonomic challenges, inadequate practical conditions, knowledge gaps, paradigm conflicts, and restrictive guidelines, hindering the provision of waterbirth services.

The midwives in this study provided euphoric descriptions of attending waterbirths described as an empowering experience where the woman gave birth instead of the baby being delivered. The ambiance in the bathroom enhanced the calming effect of water immersion. In a study conducted at the university hospital, the bathtub was scored as the most valued item in the room by the birthing women, and the way the birthing suites were organized, was of great importance to the women^24^.

The importance of knowledge in assisting waterbirths was unanimous in the interviews. Before waterbirths were accepted by the hospital, they were often carried out in secret. The knowledge on assisting waterbirths was passed on from midwife to midwife, a knowledge that was experience-based and possibly not always evidence-based. According to Weaver^25^, midwives should have knowledge about waterbirth guidelines and research to support women opting for waterbirth. As the knowledge is low and a group of midwives is reluctant to offer waterbirth, recently graduated midwives lack confidence in waterbirth. This, because waterbirth is not included in the midwifery syllabus at the local University. Consequently, midwifery students are contingent upon guidance from their clinical preceptors during practical placements to garner exposure and experience in waterbirth procedures^26^.

Hospitals organizing workshops endorses the practice and legitimizes waterbirth, and a goal to educate all midwives in waterbirth would make care equal and less dependent on the midwife assigned at birth. Ulfsdottir et al.^15^ found that many women had not in fact planned a waterbirth before the start of labor but were given the option by the midwife. Midwives influence birth mode, and the study concluded that a group of midwives are still negatively inclined.

The implementation guide^27^ of the Swedish National Board of Health and Welfare claims knowledge to be one of the success factors which correlates well with the midwives’ opinion that learning about waterbirth was crucial, and the workshops now offered impel midwives to include waterbirth in their line of work. Following knowledge, coaching and supervision must be provided in the implementation of a new process. It improves compliance and method development. In this context, the midwives at the clinic with special focus on waterbirth can obtain that role, as they support midwives in practical issues, are updated on new research and supply midwives with continuous teaching and assistance.

The Swedish National Board of Health and Welfare^27^ states that a supportive organization and an effective leadership are important factors in achieving a successful implementation. This was recognized in the interviews where the midwives’ claimed that guidelines, a diagnosis code, and supportive leadership for waterbirth helped to remove the previously perceived stigma. Managers were keypersons to spread acceptance for physiological birth within the units. Updated guidelines gave managers a legal possibility to push midwives to start using it. A snowball effect could be noted as more midwives were inclined to assist waterbirths. The routine of two midwives present at birth was seen as especially important at waterbirths for several reasons, for learning, spreading knowledge, feeling safer, and sharing a beautiful experience.

The first set of guidelines at the university hospital had five obstetricians as authors, with little or no experience of waterbirth. It is probable that the composition of authors and professions impacted its risk-oriented approach^18^. However, as the guidelines were updated and more allowing, midwives felt it was in accordance with their view of how waterbirth should be managed as a means to protect physiological birth and were regarded positively.

Increased focus on risks has permeated into the birthing rooms and has caused professional anxiety^28^. The fear of malpractice and bad outcomes has allowed the biomedical model to prevail in order to handle the evolving risk-culture. The midwives expressed a fear of increased interventions and a pathological approach on waterbirth if obstetricians got involved. Obstetricians’ involvement in normal birth creates for midwives a sense of being controlled and directed^20^. Midwives’ decision making and autonomy in handling normal birth might be impacted by this practice. However, midwives request trust from other professions in order to handle normal birth independently^29^.

As long as the concept of waterbirth is reliant upon the different hospitals, the declaration of equal care in the Patient Health Care Act^30^, which emphasizes autonomy and patient participation, is inoperative. It also hinders women’s possibilities of co-determination and obstructs midwives’ ability to work in a woman-centered way. The midwives felt split between following the Patient act and conforming to guidelines when these diverged, leading to ethical and professional stress.

### Strengths and limitations

Although the midwives in the study varied in age, occupation, and unit, they were all employed at the same hospital. Conducting a multi-center study could have resulted in a more diverse set of outcomes. Participants were recruited through self-registration, which might be particularly attractive to midwives with a positive inclination towards waterbirth compared to the broader midwifery population. However, both positive and negative aspects were presented in the results. Despite the intention for objectivity, there is the possibility of influencing participants’ discussions by the midwife having a favorable view of waterbirth. Throughout the analyses, the researchers were on guard of their own assumptions, beliefs, and presuppositions that they might apply to the study but were also aware that complete reduction is not possible.

## CONCLUSIONS

The study reveals that the midwives found both promoting and obstructing factors affecting the provision of waterbirth. The prominent factor was identified to be paradigm conflicts, with a clear distinction between a risk-focused, medicalized approach that inhibits waterbirth and a salutogenic perspective advocating for it. This dichotomy underscores the significance of the care-culture within a birthing unit and emphasizes the importance of fostering a collaborative relationship between obstetricians and midwives, with defined roles in the context of waterbirth. Furthermore, the study underscores the pivotal role of unit management in not only providing practical support but also expressing approval for a contentious practice perceived by midwives as enhancing women’s ability to experience a normal birth. These results may offer valuable insights for hospitals, both in Sweden and internationally, seeking to introduce waterbirth as an option within their maternity care services. Finally, national evidence-based guidelines are suggested to facilitate development of equitable maternity care practices.

## Data Availability

The data supporting this research are available from the authors on reasonable request.
